# Ultrafast Laser Filament-induced Fluorescence Spectroscopy of Uranyl Fluoride

**DOI:** 10.1038/s41598-018-29814-8

**Published:** 2018-08-02

**Authors:** P. J. Skrodzki, M. Burger, L. A. Finney, F. Poineau, S. M. Balasekaran, J. Nees, K. R. Czerwinski, I. Jovanovic

**Affiliations:** 1 Department of Nuclear Engineering and Radiological Sciences, University of Michigan, Ann Arbor, MI 48109 United States; 2 Center for Ultrafast Optical Science, University of Michigan, Ann Arbor, MI 48109 United States; 3 Department of Chemistry, University of Nevada Las Vegas, Las Vegas, NV 89154 United States

## Abstract

Uranyl fluoride (UO_2_F_2_) is a compound which forms in the reaction between water and uranium hexafluoride, a uranium containing gas widely used for uranium enrichment. Uranyl fluoride exhibits negligible natural background in atmosphere; as a result, its observation implies the presence and active operation of nearby enrichment facilities and could be used as a tracer for treaty verification technologies. Additionally, detection of UO_2_F_2_ has a potential application in guiding remediation efforts around enrichment facilities. Laser-induced fluorescence (LIF) has been proposed in the past as a viable technique for the detection and tracking of UO_2_F_2_. We demonstrate that ultrafast laser filamentation coupled with LIF extends the capabilities of standard LIF to enable remote detection of UO_2_F_2_. An intense femtosecond laser pulse propagated in air collapses into a plasma channel, referred to as a laser filament, allowing for the extended delivery of laser energy. We first investigate the luminescence of UO_2_F_2_ excited by the second harmonic of an ultrafast Ti:sapphire laser and subsequently excite it using the conical emission that accompanies ultrafast laser filamentation in air. We measure the decay rates spanning 4.3–5.6 × 10^4^ s^−1^ and discuss the characteristics of the luminescence for both ultrafast- and filament-excitation. Larger decay rates than those observed using standard LIF are caused by a saturated component of prompt decay from annihilation of dense excited states upon excitation with an ultrafast source. The reproducibility of such decay rates for the given range of incident laser intensities 1.0–1.6 × 10^11^ W cm^−2^ is promising for the application of this technique in remote sensing.

## Introduction

International treaties such as the recent Joint Comprehensive Plan of Action ratified in late 2015 are paramount to global nonproliferation efforts, but they present numerous challenges to their enforcement. It has been generally accepted that novel, innovative technologies could significantly benefit the verification of those treaties. One of the cornerstone activities in nuclear proliferation is the enrichment of uranium. Although most countries agree to monitoring of their enrichment activities by the International Atomic Energy Agency, a relatively small, clandestine facility based on a technology such as centrifuge enrichment can remain undetected and pose a threat to global nonproliferation efforts^1,2^. Therefore, the development and use of contemporary technologies for detection of undeclared enrichment activities is crucial for maintaining nuclear security.

Uranium enrichment processes such as gaseous diffusion and gas centrifuge use uranium hexafluoride (UF_6_), a gas which when exposed to water (such as water vapor in air) forms hydrofluoric acid (HF) and uranyl fluoride (UO_2_F_2_):

1 $${{\rm{UF}}}_{\mathrm{6(}g)}+2\,{{\rm{H}}}_{2}{{\rm{O}}}_{(g)}\to {{\rm{UO}}}_{2}{{\rm{F}}}_{\mathrm{2(}s)}+4\,{{\rm{HF}}}_{(g)}\mathrm{.}$$

Of those two compounds, UO_2_F_2_ exhibits a near-zero natural background in the atmosphere. The presence of UO_2_F_2_ in the atmosphere, therefore, indicates the presence of nearby enrichment facilities, from which UF_6_ leaks at low rates. Hence monitoring UO_2_F_2_ emitted from centrifuge or supporting facilities which process UF_6_ is a viable approach to detect undeclared enrichment activities^2^. Moreover, detection and careful audits of UO_2_F_2_ concentration may have applications in safeguards, for example by guiding the remediation efforts around enrichment facilities.

Detection of UF_6_, along with the other byproducts of enrichment, namely UO_2_F_2_ and HF, has been proposed via several techniques, including remote LIDAR^3^, air sampling methods that use particle filtration^2,4^, and air sampling with laser ablation-laser absorbance ratio spectrometry (LAARS)^5,6^, laser-induced breakdown spectroscopy (LIBS)^7^, and laser-induced fluorescence (LIF) spectroscopy^6,8–11^. UO_2_F_2_ proves the most promising tracer due to its chemical stability. It remains in an aerosol state on order of days (limited mainly by its solubility in water)^2^, unlike UF_6_. In addition, the UO_2_F_2_ natural background is negligible when compared to HF. Limitations of the detection techniques proposed to date include their limited range in the case of air sampling methods and standard LIBS, long measurement durations for air sampling and swipe methods, and the loss of information from dissociation of the UO_2_F_2_ compound upon breakdown using LIBS. Nontrivial analysis of the stoichiometry in laser-produced plasmas and consideration of the natural backgrounds of oxygen and fluorine species would be required to infer the presence of UO_2_F_2_ when techniques such as LIBS are used.

In contrast, the approach in which luminescence is observed without inducing optical breakdown carries significant benefits. Unlike optical breakdown, which typically dissociates larger molecules such as UO_2_F_2_, it preserves the structure of the compounds, which may itself be a component of the characteristic signature. Further, less energy is required to excite vibrational modes of molecules and the luminescence is typically longer lived, ${\mathscr{O}}({\rm{ms}})$^10^, in comparison to LIBS signal lifetime, ${\mathscr{O}}$(µs)^7^. In the 1980s, Chimenti and co-workers proposed the remote monitoring of luminescence of uranyl for ore prospecting using LIF^12,13^. Employing LIF to distinguish among the various uranyl compounds has been extensively researched in the past^8,9,11,14–33^. In this work, we measure the temporal and spectral characteristics of luminescence of UO_2_F_2_ following its excitation by radiation produced from femtosecond (fs) laser filaments and demonstrate that this method can be used for remote detection of UO_2_F_2_.

Intense, ultrafast laser pulses propagated in air undergo Kerr self-focusing due to an intensity-dependent change in refractive index Δ*n* = *n*_2_*I*, where *n*_2_ is the Kerr index associated with the third-order nonlinear susceptibility of the medium, with magnitude ${\mathscr{O}}{\mathrm{(10}}^{-19}{{\rm{cm}}}^{{\rm{2}}}\,{{\rm{W}}}^{-{\rm{1}}})$ in air^34^ and *I* is the laser intensity. The focused pulse excites and ionizes the medium, forming a plasma which acts jointly with self-focusing to transport the laser pulse energy through a long, narrow channel. Filamentation has been shown to extend the propagation distance of energetic femtosecond laser pulses up to the order of 1 km while maintaining a tight focus^35–38^. The laser radiation emitted from the end of the filament plasma exhibits an increased angular distribution as well as spectral broadening and temporal compression^39,40^; the lower intensity of this divergent *conical emission* compared to the intensity of the filament core in the plasma channel makes it a suitable candidate for laser-induced fluorescence without optical breakdown of the target. Several works previously demonstrated remote sensing that combines filamentation and LIDAR of molecular pollutants such as halocarbons^41^, ethanol as a surrogate for other polluting hydrocarbons^42^, CH_4_, C_2_H_2_, C_2_H_4_, ethanol vapor, and smoke^43^. We explore the potential to excite the luminescence of UO_2_F_2_ following optical filamentation in air using the second harmonic $({\lambda }_{0}\sim 400\,\mathrm{nm})$ of laser pulses produced by a Ti:sapphire chirped-pulse amplification system. The second harmonic of the Ti:sapphire laser (400 nm) is well-coupled to the absorption spectrum of uranyl compounds, yielding a luminescence band between 450 and 600 nm^9^.

## Results and Discussion

### The signature of uranyl fluoride upon excitation with a frequency-doubled Ti:sapphire ultrafast laser

**Figure 1:**
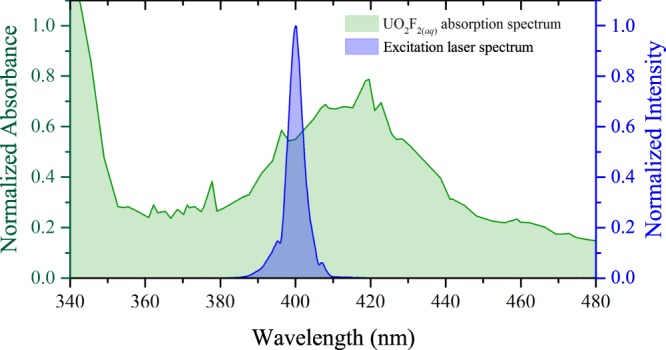
Measured absorption spectrum of UO_2_F_2_ in solution using UV/VIS spectrophotometer (green); incident laser spectrum (blue).

**Figure 2:**
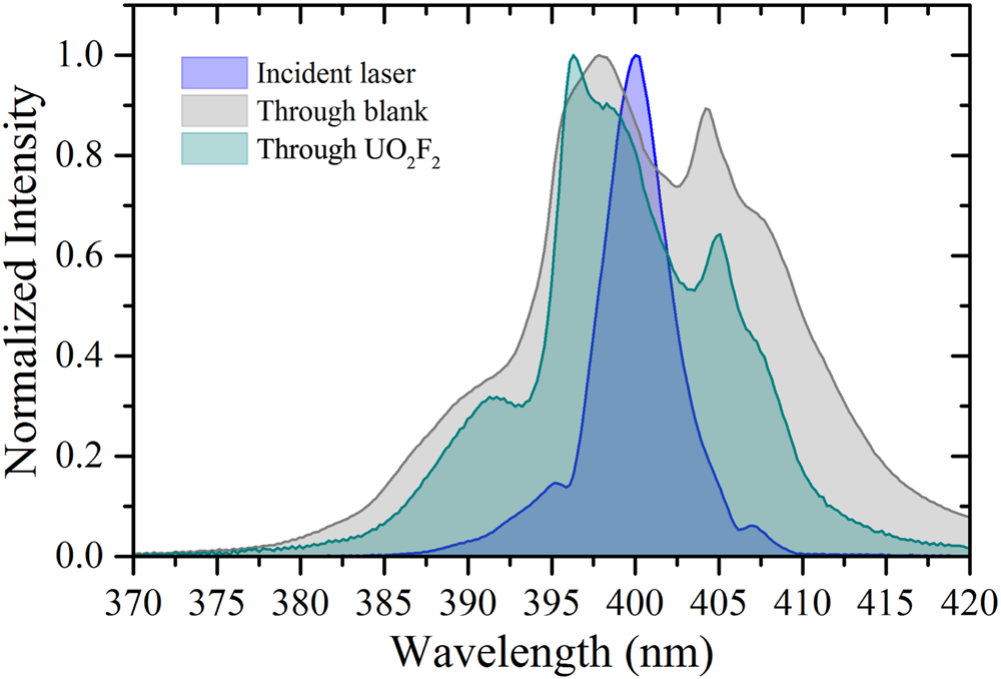
Laser spectrum after passage through blank HF sample (gray) and through UO_2_F_2_ in solution (cyan) compared to incident laser spectrum (blue).

Understanding not only the time-integrated, but also the time-dependent characteristics of the luminescence spectrum is important for applications in remote sensing, in which distance, collection efficiency, and backgrounds affect the detection limits. For example, the unique decay rate of the luminescence provides an additional source of information, which may help distinguish signal from various backgrounds that carry different time characteristics. The absorption spectrum of uranyl compounds peaks near 420 nm, spanning ∼350–500 nm^9,44,45^. Figure 1 shows the incident excitation laser spectrum overlaid on a normalized UV/VIS spectrophotometer measurement of the absorption spectrum for the UO_2_F_2_ solution. The broad spectrum of the frequency-doubled Ti:sapphire laser is near the peak of the absorption spectrum. Figure 2 shows the laser spectrum transmitted through the blank solution as well as that transmitted through the UO_2_F_2_ solution. The laser spectrum through the analyte shows preferential absorption toward the longer wavelengths, approaching the absorption peak at ∼420 nm^44,45^. The broadening of the laser spectrum observed through the blank sample results from the effects of self-phase modulation throughout the propagation path of the intense laser pulse.

The characteristic luminescence of uranyl compounds typically exhibits four to six band structures arising from magnetic dipole emission from the lowest triplet ^3^Δ_*g*_ excited state to five vibrational levels in the singlet ${}^{1}{\rm{\Sigma }}_{g}^{+}$ ground state^9,45^. The emission, therefore, is primarily phosphorescence, involving inter-system crossing which yields a longer-lived decay. The decay of luminescence signal *I*_l_(*λ*, *t*) is exponential in time and contains the contributions of several emitting pathways:

2 $${I}_{{\rm{l}}}(\lambda ,t)=\sum _{i}{A}_{i}{S}_{i}(\lambda )\,\exp (\,-\,{\gamma }_{i}t),$$

where *A*_*i*_ and *S*_*i*_(*λ*) describe the strength and spectral band shape, respectively, while *γ*_*i*_ is the characteristic decay rate for the uranyl complex *i*^46^. Beitz *et al*.^10^ measured a characteristic decay rate ∼6 × 10^3^ s ^−1^ via 337-nm long-pulse laser excitation, resulting in observed lifetimes between ∼100 μs and ∼300 μs for uranyl ion concentration of 1.3 × 10^−5^ M. Budylin *et al*.^46^ conducted a comprehensive study of the decay rates at varying ns-laser intensities in the range 10^6^–10^8^ W cm^−2^ for different uranyl complexes. Budylin *et al*. further discussed the contribution of a rapidly decaying, approximately exponential component for greater laser intensities, yielding a greater overall decay rate: increasing laser intensity also increases the density of excited states, causing a substantial rate of excited state annihilation, as opposed to the slower phosphorescence de-excitation^46^. We compare the nature of the luminescence excited by an ultrafast source with intensity ∼10^11^ W cm^−2^.

**Figure 3:**
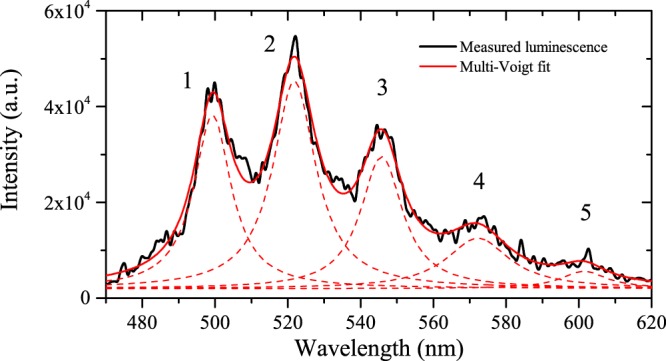
Example of a multi-Voigt fit (red) to measured luminescence (black) with 1-mJ incident energy at delay 0 s.

**Figure 4:**
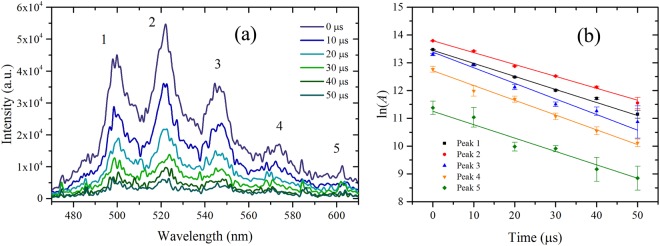
(**a**) Time-dependent luminescence of UO_2_F_2_ in solution excited by 1-mJ incident energy accumulated for 200 laser shots. (**b**) Logarithm of peak area *A* determined by fitting data with multi-Voigt algorithm as a function of time; the linear fits determine the decay constant *γ* for each peak (Table 2).

The luminescence exhibits five broad peaks, labeled 1–5 in Fig. 3. We compare the decay rates for each major feature in the luminescence spectrum in order to identify behavior that may be useful for remote sensing, such as individual peak decay rates. The peaks are fit with a multi-Voigt^44^ profile to account for the natural (Lorentz)^45^ shape of the transitions as well as instrumental (Gaussian) broadening using least squares with a Nelder-Mead simplex algorithm to optimize fit parameters. Figure 3 shows an example fit for luminescence excited by 1-mJ incident laser energy with gate delay of 0 μs and gate width of 10 μs. Adjusted *R*^2^ values range from 0.98 for data at earlier delays to 0.82 for data at later delays. Table 1 contains peak centroids and widths determined from the fits averaged for five long-gate (100 μs) measurements with gate delay of 0 µs and incident laser energy of 1 mJ. Figure 4(a) shows the time-dependent decay of the luminescence spectrum with 10 μs delay increments and 10 μs gate width at each delay; Fig. 4(b) shows the logarithm of the individual fitted peak areas as a function of time. The data is fitted with a linear model to determine the rate of decay *γ* shown for each peak in the legend. Adjusted *R*^2^ values are greater than 0.96 for each model. Decay rates are tabulated in Table 2. Notably, decay rates are similar among the five observed peaks, implying similar probabilities of transition into each of the vibrational levels in the ground state. The measured decay rates are in the range of 4.3–5.6 × 10^4^ s^−1^ and are an order of magnitude larger than those determined by Beitz *et al*.^10^, who used a longer pulse duration laser. These results are more comparable to those presented by Budylin *et al*.^46^ for higher intensities, *γ* = (9.2 ± 2.7) × 10^4^ s^−1^ for intensity of 1.8 × 10^7^ W cm^−2^. We further investigate the effects of laser intensity in the regime ${\mathscr{O}}$(10^11^ W cm^−2^).

**Table 1:** Luminescence peak centroids and widths determined from multi-Voigt fit of data averaged for five long-gate (100 μs) measurements with gate delay of 0 μs and incident laser energy of 1 mJ.

Peak label	Centroid (nm)	Width (FWHM, nm)
1	500.25 ± 0.11	13.21 ± 0.59
2	521.56 ± 0.21	13.45 ± 0.66
3	546.16 ± 0.39	16.29 ± 0.70
4	572.65 ± 0.22	17.31 ± 0.94
5	599.74 ± 0.69	20.64 ± 3.68

**Table 2:** Estimated decay rates excited by 1-mJ incident laser energy and filament-excited decay rates for 1.6-mJ energy before filamentation and 1-mJ incident on the front face of the sample.

Peak	Decay rate, *γ*	Filament-excited decay rate, *γf*
	(×10^4 ^s^−1^)	(×10^4 ^s^−1^)
1	4.7 ± 0.1	4.7 ± 0.2
2	4.3 ± 0.1	4.4 ± 0.2
3	5.6 ± 0.5	4.7 ± 0.2
4	5.3 ± 0.2	4.6 ± 0.4
5	4.8 ± 0.8	5.5 ± 0.4

**Figure 5:**
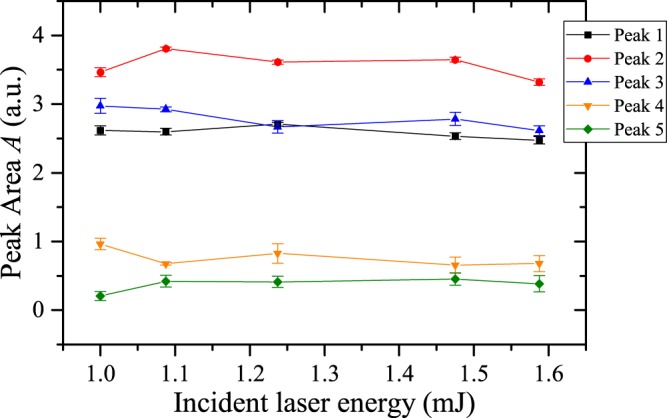
Peak area for each of the five major peaks observed in the luminescence spectrum for varying incident laser energies; peaks are labeled in Fig. 3.

Figure 5 demonstrates that saturation of the luminescence occurs over the range of laser intensities of 1.0–1.6 × 10^11^ W cm^−2^. Excitation occurs on the order of several picoseconds, so that interaction with an ultrafast (femtosecond) laser results in a nearly instantaneous production of a large number of excited states, increasing the probability of interactions between complexes in the excited states that result in annihilation, as discussed by Budylin^46^. The observed signal saturation over this range of laser intensities is attributed to the local density of excited states (in space and time) reaching a maximum for the given analyte concentration. Further work is necessary to confirm that triplet-triplet annihilation is more prevalent for femtosecond-excitation and to explore the effect of concentration and laser intensity on the occurrence of this phenomenon in more detail.

### Excitation of uranyl fluoride with the conical emission following optical filamentation

The incident laser energy also plays an important role, since filamentation is a threshold phenomenon: the onset of self-focusing occurs when the power reaches the level referred to as critical power, *P*_*cr*_. The critical power derived for a cw Gaussian beam is^47^

3 $${P}_{cr}=\frac{3.79{\lambda }^{2}}{8\pi {n}_{0}{n}_{2}},$$

where *n*_0_ and *n*_2_ represent the refractive and Kerr indices of the medium at a wavelength *λ*, respectively. The calculated critical power for a 400-nm beam propagating in air is ∼0.5 GW. Incident peak powers greatly exceeding the critical power yield the formation of several intense filament cores, which are surrounded by a large energy reservoir^34^. This regime, in which multiple filamentation is observed, is the working range for long-distance applications because the propagation distance of the filament increases with incident laser power. Hence, we perform experiments spanning (40–60) × *P*_*cr*_, which ensures the relevance of outcomes for future field experiments.

**Figure 6:**
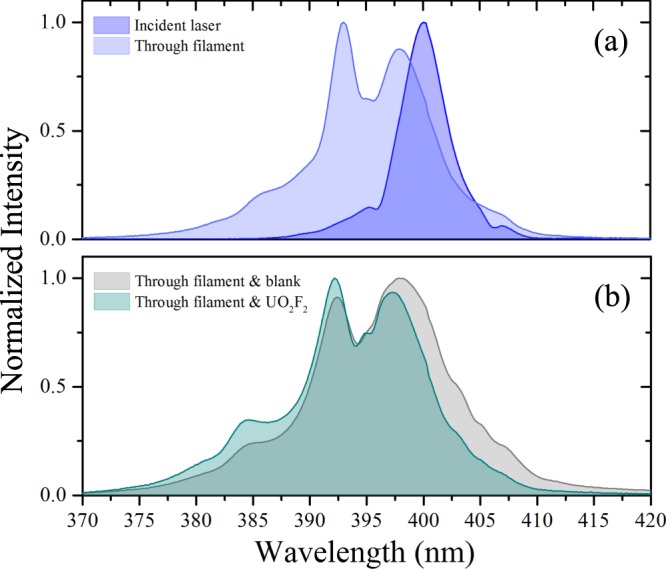
(**a**) Comparison of the laser spectrum after filamentation (lighter blue) with the incident laser spectrum (darker blue). (**b**) Comparison of the spectrum after the filament transmitted through the blank HF sample (gray) with the UO_2_F_2_ in solution (cyan).

**Figure 7:**
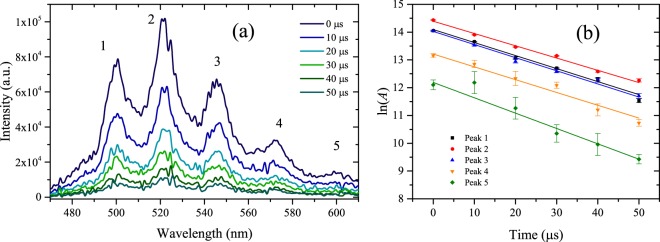
(**a**) Time-dependent luminescence of UO_2_F_2_ in solution excited by 1.6-mJ incident energy before filamentation and 1-mJ measured at the front face of the sample accumulated for 300 shots. (**b**) Logarithm of peak area *A* determined by fitting data with multi-Voigt algorithm as a function of time; the linear fits determine the decay constant *γ*_*f*_ for each peak (Table 2).

Finally, we demonstrate the ability to excite luminescence with radiation following filamentation. The incident laser energy is 1.6 mJ (60 × *P*_*cr*_), and the transmitted energy through the plasma measured at the front face of the sample is 1 mJ. The divergent radiation following the filament plasma is commonly referred to as conical emission^39,40^; this term encompasses also the guided laser radiation. Figure 6(a) shows the laser spectrum measured after the filament plasma at the front face of the sample. The detailed origin of conical emission and the correspondent spectral broadening and increased angular distribution is still being debated; candidate mechanisms, outlined by Maioli *et al*.^39^ and Béjot *et al*.^40^ and references therein, include Cherenkov radiation, self-phase modulation, and other high-order Kerr effects. We observe similar preferential absorption of the longer wavelengths approaching the peak of the absorption spectrum in Fig. 6(b). Moreover, we observe similar decay rates as with ultrafast excitation, as shown by Fig. 7; the measured filament-excited decay rates *γ*_*f*_ for each of the five major peaks of the luminescence are compared to those observed by ultrafast excitation in Table 2. Evidently, the signal remains quenched despite the change in incident laser spectrum among other laser parameters associated with the conical emission. This result demonstrates the ability to reproduce the signal observed by ultrafast excitation with the conical emission following filamentation.

## Conclusion

Uranyl fluoride, which can form in the reaction between uranium hexafluoride leaking from a uranium enrichment facility and water vapor from the atmosphere, could be a powerful indicator of an active uranium enrichment process. We investigated the luminescence of UO_2_F_2_ excited by a frequency-doubled ultrafast Ti:sapphire laser in order to subsequently combine the standard LIF technique with optical filamentation and enable remote sensing capability. UO_2_F_2_ is soluble in water, limiting its survivability as an aerosol to the order of days^2^; hence the optical signal generated by femtosecond- and filament-excitation of aqueous uranyl fluoride is of more general value and serves as the basis for future detection in more realistic field scenarios. We measured decay rates in the range of 4.3–5.6 × 10^4 ^s ^−1^ for both ultrafast- and filament-excitation. The relatively fast decay rate in comparison to that previously reported by Beitz *et al*.^10^ and Budylin *et al*.^46^ can be explained by a significant prompt component originating from the annihilation of triplet excited states of uranyl as opposed to de-excitation from the triplet excited state to singlet ground states, which is a slower, phosphorescence decay. Furthermore, quenching of the luminescence signal at increased laser intensity implies a saturated density of excited states, which produces a maximum decay rate by the aforementioned mechanism. The reproducible total emission irrespective of incident laser intensity as well as the reproducible decay rate of ∼10^4 ^s^−1^ for the given range of intensities with femtosecond-excitation bodes well for remote measurements because a consistent signal may be observed. However, further work is necessary to determine the limiting analyte concentration and laser intensity characteristic for this quenching regime; in the case of a saturated density of excited states, the measured decay rate may be correlated to analyte concentration. Ultimately, we excited the luminescence of UO_2_F_2_ with the conical emission following optical filamentation and observed emission similar to the case of ultrafast excitation. We show that the second harmonic of an amplified Ti:sapphire laser, which is easily produced with high efficiency, as well as the conical emission following filamentation of the second harmonic are optimal matches for the absorption wavelengths of uranyl fluoride. Moreover, the delivery of an intense nanosecond laser excitation source is limited to shorter distances due to the size of optics that is required to focus it. In contrast, the proposed filament-excitation of uranyl fluoride overcomes this limitation and has been shown in previous work to extend the delivery of an intense excitation source to distances on order of kilometers.

## Materials and Methods

### Sample Preparation

UO_2_F_2_ samples were prepared at the University of Nevada Las Vegas in a radiochemistry laboratory designed for chemical synthesis using efficient HEPA-filtered fume hoods and glove boxes and following locally approved radioisotope handling and monitoring procedures.

Anhydrous UO_2_F_2_ was prepared according the method outlined in^48^. In this procedure, UO_2_(NO_3_)_2_6 H_2_O was initially converted to UO_4_2 H_2_O by treatment with conc. HNO_3_ and H_2_O_2_. The resulting UO_4_2 H_2_O was dissolved in 24% HF in a Teflon flask. The flask was connected to a Schlenk line and treated at 110 °C for 1 hr under flowing argon. After this time, a resulting yellow solid (UO_2_F_2_) was obtained, placed in a desiccator over conc. H_2_SO_4_ for one week before being dissolved in 3 mL 0.05 M HF/0.05 M KF to make a 0.05 M UO_2_F_2_ solution, and shipped to the University of Michigan for spectroscopy.

### Ultrafast laser- and filament-induced fluorescence spectroscopy of uranyl fluoride

**Figure 8:**
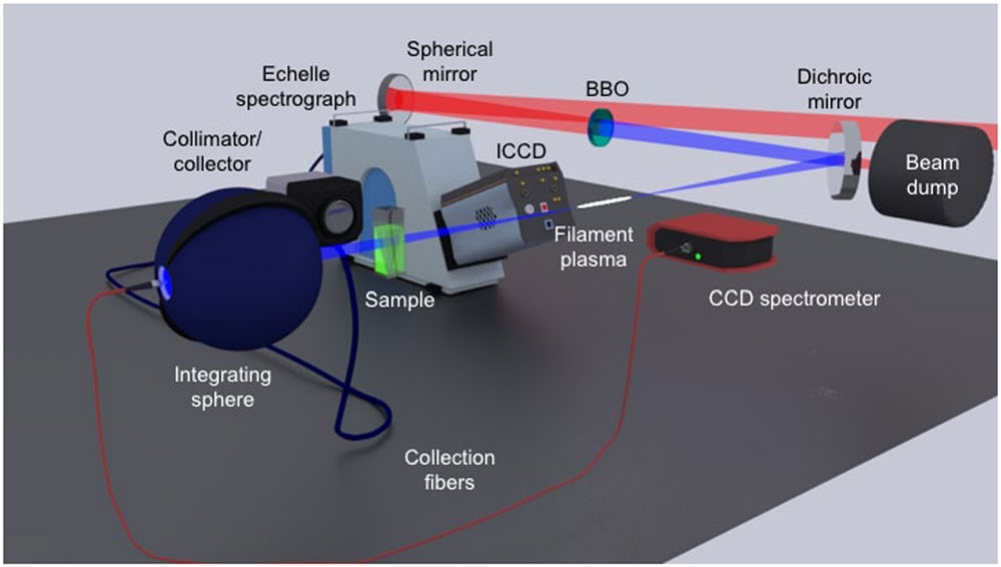
Experimental schematic for filament excitation. The incident 800-nm pulses are focused with an *f*/40 spherical mirror through a 100 μm-thick BBO crystal to create 400-nm pulses; residual 800-nm pulses are removed using a high-pass dichroic mirror. The filament plasma forms near geometrical focus of the spherical mirror; the conical emission expands after the filament plasma and excites the 0.05 M UO_2_F_2_ solution, placed 30 cm after the geometrical focus. Sample luminescence is collected with an *f*/2 collimator and transported through a 400-μm diameter optical fiber into an Echelle spectrograph coupled to an ICCD detector. Optical spectra are collected using an integrating sphere and directed into a compact CCD spectrometer via optical fiber.

The experimental schematic is shown in Fig. 8. The laser used in this study is the custom-built Lambda Cubed (*λ*^3^) Ti:sapphire-based two-stage chirped-pulse amplification system at the University of Michigan. Operating conditions for our experiment include the pulse duration of 50 fs, repetition rate of 80 Hz, and pulse energies spanning 1–1.6 mJ. All experiments were performed in air. The second harmonic is generated using a 100-μm thick *β*-Ba(BO_2_)_2_(BBO) crystal. The pulse duration of generated second harmonic is calculated^49^ to be 41 fs. Residual 800-nm light was removed with a high-pass dichroic mirror, ${\mathcal R}$(800 nm) < 0.2%. The analyte was UO_2_F_2_ (0.05 M) dissolved in 3 mL 0.05 M HF/0.05 M KF contained in a 1 cm × 1 cm PMMA cuvette, ${\mathcal L}$(>300 nm) > 80%. Transmission measurements were compared to a *blank* solution of 0.05 M HF/0.05 M KF contained in an identical cuvette. The total light path for each sample and blank included 2 mm of PMMA and 8 mm of solution. The measured transmission efficiency through the blank sample was $(8\overline{0}\,\pm \,3)$% (transmitted laser energy through blank normalized to incident laser energy); losses occurred primarily due to reflection at each interface along the light path. Filamentation occurred after focusing the laser beam with a silver coated, 50-mm diameter, 1-m focal length spherical mirror (*f*/40). The observed filament plasma length was ∼4 cm near the geometrical focus of the spherical mirror. For filament excitation measurements, the sample was placed 30 cm following geometrical focus of the spherical mirror. Laser spectra were recorded with an integrating sphere and time-integrated over 1 ms using a compact CCD spectrometer (CCS200, Thorlabs). Sample luminescence was collected with a *f*/2 collimator (CC52, Andor) coupled into a 400-μm diameter optical fiber. The detector system is an Echelle spectrograph (ME5000, Andor) coupled to an ICCD (iStar 334T, Andor). The spectrograph slit dimensions are 50 μm × 50 μm (resolving power 5000). Spectra were accumulated for several shots in order to improve signal-to-noise. The detector system was calibrated with a mercury-argon lamp (Pen Light, Oriel) and radiometric source (DH-2000, Ocean Optics). Each spectrum is normalized to the transmission function of the system.
